# Acknowledgment to the Reviewers of *Micromachines* in 2022

**DOI:** 10.3390/mi14020262

**Published:** 2023-01-19

**Authors:** Micromachines Editorial Office

**Affiliations:** MDPI AG, St. Alban-Anlage 66, 4052 Basel, Switzerland

High-quality academic publishing is built on rigorous peer review. *Micromachines* was able to uphold its high standards for published papers due to the outstanding efforts of our reviewers. Thanks to the efforts of our reviewers in 2022, the median time to first decision was 14 days and the median time to publication was 31 days. Regardless of whether the articles they examined were ultimately published, the editors would like to express their appreciation and thank the following reviewers for the time and dedication that they have shown *Micromachines*:
A. B. M. Harun Ur RashidMandeep Singh Jit SinghA. J. S. (Sam) SpearingManfred KrautA. Kerem UguzManfredo G. GuilizzoniAamer NazirMangey Ram NagarAamir AliManh-Cuong NguyenAaron J. PungManibalan KesavanAaron OhtaManikandan SanthanamAatif Ali ShahManish MukhopadhyayAbas SabouniManish SharmaAbbas HaddadiMan-Kay LawAbbas PanahiMannix P. BalanayAbbas RezaeeManoharan PremkumarAbbas RohaniManoj KumarAbd Rashid Bin Mohd YusoffManoj SaxenaAbdalrahman QubaaManojit PustyAbdelilah AsserghineManuela PanoiuAbdelkader AbderrahmaneMaomao ChenAbdelkader AissatMar ÁlvarezAbdelkader ShaabanMaral AnsariAbdellah BoulouzMarat MinlebaevAbdel-Nasser SharkawyMarc Christopher WurzAbderrahmane AissaMarc OlanoAbderrazak BoutramineMarcel SchuckAbdollah AllahverdiMarcello BertoAbdul Azeez Abdu AliyuMarcello CasaAbdul Hadi Bin AzmanMarcello IasielloAbdul Samad KhanMarcin GnybaAbdulkafi SaeedMarcin GołąbczakAbdullah N. AlodhaybMarcin JanickiAbeer SalahMarcin JesionekAbel MorenoMarcin NabiałekAbhay KochharMarcin SzczechAbhijit ChatterjeeMarcio Bacci Da SilvaAbhishek KumarMarcio Bender MachadoAbhishesh MehataMarcio De GodoyAbil S. AsvarovMarco AllioneAbinash GayaMarco BarbatoAbtin AtaeiMarco CarlottiAbu SufianMarco CarusoAda-Ioana BuneaMarco GandolfiAdam AbramowiczMarco GirolamiAdam GlowaczMarco HuberAdam KawalecMarcos F. S. TeixeiraAdam KhanMarcos MarcosAdam LeckoMarek KalenikAdam MiranowiczMarek KieliszekAdam NarbudowiczMarek KocikAdeeb SalhMarek KorzeniewskiAdele De NinnoMarek PokornyAdem OzcelikMarek SzkodoAdina NegreaMarek Vrabel′Adithya SridharMargarida BritoAditya S. YerramilliMargarida RibeiroAdnan MahmoodMargarita A. KhimichAdnan ShakoorMargo StaruchAdnane NoualMaria DimakiAdolfo R. LopezMaria FreitasAdrian GligorMaría I. Álvarez EchazúAdrian J. T. TeoMaría José Bautista-LlamasAdrian Krzysztof AntosikMaria K. RybarczykAdriano BarraMaria KoskinopoulouAdriano VernaMaría Liz CrespoAdrien MorelMaría Moreno-GuzmánAdrienne MinerickMaria PantanoAdy SuwardiMaria RaposoAfshin AbrishamkarMaria Rita CiteroniAgarwal Ambuj KumarMaria RomodinaAgata Lisinska-CzekajMaria WiesławaAgathoklis A. KrimpenisMarian WnukAgnes PurwidyantriMariana DalarssonAgnieszka BrzózkaMariangela QuartoAgnieszka PodwinMariella SärestöniemiAgnieszka PregowskaMarija MilanovićAgnieszka SkoczylasMarija Radmilović-RadjenovićAgnivo GosaiMarijo BuzukAgustin L. Herrera-MayMarin KovačićAhmad AlShwawraMarina RumyantsevaAhmad HassanMarina ShavelkinaAhmad JabbarzadehMario Alberto García RamírezAhmad OmarMario DeMiguel-RamosAhmed A. IbrahimMario Ricardo Gongora-RubioAhmed AlfadhelMario RothbauerAhmed ElkaseerMario Siciliani De CumisAhmed Hisham Eissa MorshedMario VranješAhmed JalalMarioara AvramAhmed Jamal Abdullah Al-GburiMarion LeiboldAhmed Kadhim HusseinMarius Andrei AvramAhmed M. AttiyaMarius BumbacAhmed MaamounMarius PrelipceanuAhmed SabryMariusz DejaAhmed ShakerMariusz TryznowskiAhmet Emin TopalMarjan MernikAhmet Fatih TabakMark A. BerhowAhsan AltafMark A. LantzAida NaghilouMark Cronin-GolombAidin Bordbar-KhiabaniMark MelnykowyczAini Zuhra Abdul KadirMarkos PetousisAi-Xiang DingMarkus LahikainenAjay Vikram SinghMaros SmondrkAjay VyasMarta I. HernándezAjit BeharaMarta SzekalskaAkhilesh JaiswalMartin De BeerAkhilesh PathakMartín G. FrixioneAkihiro IsozakiMartin RichterAkilarasan MuthumariappanMartin SharpAkiomi UshidaMartin StimpfelAkitaka ItoMartin V. IvanovAlaa AttarMartine PrévostAlaa ZakyMartino AldrigoAlbert Cisquella SerraMárton MátéAlbert E. PattersonMarvin ChengAlbert LeeMaryam HajiabbasAlbert Wen Jeng HsueMarzena LachowiczAlberto AloisioMasahiko YamaguchiAlberto GregoriMasahisa FujinoAlberto Martín-PérezMasanobu MiyataAlberto T. PérezMasanori HaraAldo De SabataMasashi IkegamiAlejandro Valencia-AriasMasato TanakaAlejandro ZacaríasMasaya IchimuraAleksandr PoliakovMasoud MadadelahiAleksandr S. LozhkomoevMasoud ShekargoftarAleksandra RanitovićMassimiliano LabardiAleksandrs MarininsMassimo MarielloAleksei V. AlmaevMassimo MastrangeliAleksey MaksimkinMassimo SpinaAleksey S. AnuchinMassimo V. FischettiAlena NastulyavichusMatej SulitkaAleš ChválaMateusz KuklaAlessandra BiancolilloMateusz PasternakAlessandra ZizzariMateusz ZelentAlessandro GabrielliMatheus S. XavierAlessandro PaghiMathew G. PelletierAlessandro SchiaviMathias BusekAlessandro TestaMatin TorabiniaAlessio BuzzinMatjaž KristlAlexander Christantho BudimanMatthew SpencerAlexander DarinskiiMatthias RädleAlexander G. DvoretskyMatthieu LancryAlexander GagarinMauro Callejas CuervoAlexander GottwaldMauro TortelloAlexander J. C. KuehneMaxim GorkunovAlexander KukaevMaxim RakhlinAlexander M. LererMaxim TopchiyAlexander MuravskyMayank SrivastavaAlexander S. MelnikovMazeyar Parvinzadeh GashtiAlexander S. SobolevMaziar HeidariAlexander ZhbanovMaziar YazdaniAlexandr SadovnikovMbaye DioufAlexandre ChicharoMd Abu ZahedAlexandros PitilakisMd Farhadur RezaAlexey BeskopylnyMd Junayed HasanAlexey G. TemiryazevMd Rasedul IslamAlexey VereschakaMd SalauddinAlfa SharmaMd SharifuzzamanAlfio BattiatoMd. Alamgir HossainAlfio Dario GrassoMd. Mahbub HossainAlfonso Gómez-EspinosaMd. Mokter HossainAlfredo LanzaroMd. Nizam UddinAli DadMd. Salauddin RaselAli Dehghan FiroozabadiMd. Samsuzzaman SobuzAli Ebrahimi-MamaghaniMd. ShahjalalAli ElrashidiMd. Tanvir HasanAli HassanMehdi BahiraeiAli J. ChamkhaMehdi HosseinzadehAli KhalfallahMehdi SafariAli KosarMehebub AlamAli MansoulMehmet AydinAli MirzaeiMehmet EgilmezAli RoshanghiasMehmet L. YolaAli ShukurMehmet PolatAli Turab JafryMeisam AsgariAli ZolfagharianMeiyong LiaoAliasghar AjamiMelania TeraAlina Adriana MineaMelina P. IoannidouAlireza BabaeiMeltem ElitasAlireza Mohammad KarimMenake PiyasenaAlireza R. KhosroshahiMeng SunAliza Aini Md RalibMeng ZhangAllison Hess-DunningMeng-Fang LinAlok Kumar DasMeng-Hua YenAlphonsus NgMengjing WangÁlvaro Romero-CalvoMengju LinAlwin PouloseMenglei HuAmado Velazquez-BenitezMengqiang ZhaoAman DurejaMengxia LiuAman MahajanMengye WangAmanpreet KaurMeng-Yun ChungAmarajothi DhakshinamoorthyMercedes Rivas-LasarteAmelie HagelauerMerih PalandokenAmer CharbajiMesut YurukcuAmin FatoniMetin AktasAmin Hamed MashhadzadehMeysam Akbari MasonAmin RabieiMeysam Sharifzadeh MirshekarlooAmir ElzwawyMichael D. GrayAmir HatamieMichael DossisAmir Hossein BehbahaniMichael HeiseAmir KhesroMichael KovalevAmirmostafa AmirjaniMichael S. GerltAmirreza AghakhaniMichael SemenovAmirreza KhodadadianMichael WehnerAmirsalar KhandanMichał BoćkowskiAmit ChhabraMichal BoreckiAmit PanwarMichał CiemałaAmit PrabhakarMichal KrupinskiAmit RaiMichal KrysztofAmit SharmaMichal SzermerAmitav TikadarMichał WojasińskiAmjad IqbalMichel DevelAmjad IslamMichele BassoAmmar OdehMichele BonninAmr KaoodMichèle GouiffèsAmrita DeyMiguel A. Quetzeri-SantiagoAmy J. MollMiguel Angel Luque-NietoAn Eng LimMiguel Ángel Mateos TimonedaAna Carolina Mendes HackeMiguel Díaz-RodríguezAna Díaz-FernándezMiguel PincheiraAna Maria RochaMiguel XavierAna Raquel Santa-MariaMihaela CirlugeaAna-Maria GurbanMihaela IonescuAna-Maria IordacheMihaela OlaruAnand Mohan ShrivastavMihaela Raluca CondruzAnandram VenkatasubramanianMihai A. GîrțuAnant AgarwalMihai DudutaAnanthakumar RamadossMihai OaneAnargyros BaklezosMihaiela IliescuAnastasia KoivikkoMihail Lucian PascuAnastasios KoutinosMikhail A. ArtemovAnatoliy RinkevichMikhail A. Eron′YanAnatoly MitrofanovMikhail A. Il′yushinAnderson Wedderhoff SpenglerMikhail BasarabAndoni BeriainMikhail KotovAndra Naresh Kumar ReddyMikhail P. KozochkinAndras SafticsMikhail SalinAndré MartinMikhail SemenovAndrea G. Martinez-LopezMikhail ShamoninAndrea González-MontoroMikhail V. GolubAndrea MainardiMikyung ShinAndrea MichelettiMilan GnjatovićAndrea OrsiniMilan ZeljkovicAndrea PastoreMilena KubišováAndrea PinnaMiłosz GrodzickiAndrea RavasioMilton ChaiAndrea RiaMin Chul ShinAndreas BahrMin Ju KimAndreas RosenkranzMin YangAndreas WeberMin YuAndreas WinklerMina HoorfarAndrei EgorinMinas StylianakisAndrei SirenkoMin-Chul LeeAndrei TurutinMinfang ZhangAndrew J. HarvieMing Tzer LinAndrey A. KuznetsovMing-An ChungAndrey A. SamokhvalovMingbo PuAndrey AltynnikovMingchang ChihAndrey FilippovMing-Cheng KaoAndrey G. AltynnikovMingfeng WangAndrey PryamikovMing-Fong TsaiAndrey SarikovMinggang XiaAndrey V. FilippovMinghao NieAndrey V. SvalovMinghao ZhaoAndrey Yu. KlokovMinghe ShanAndrey ZhirnovMing-Hsien HsuehAndrius ŽemaitisMinglei QuAndrzej MikułaMingliang JinAndrzej PacanaMingliang ZhangAndżelika KrupińskaMing-sheng XuAnees AbbasMing-Shyan WangAneta HapkaMingtao WangÁngel Serrano-ArocaMingxiang LingAngela BaracuMingxiao LiAngela Di VirgilioMingyuan ChenAngela StortiniMingze SunAngeliki TserepiMinh Long HoangAngelito SilverioMinh Phung VanAngkoon PhinyomarkMinori ShirotaAnh H. NguyenMinoru ShinoharaAnh My ChuMinseok ChoiAnhu LiMinseop SongAniket MahantiMinsoo KimAnimesh Kumar BasakMinsu JungAnirban KarmakarMiodrag ZivkovicAnirban SinhaMircea DragomanAniruddha MondalMirko MaturiAnish ShenoyMiroslav DusparaAn-Jye TzouMirza RubelAnkur GuptaMo HuangAnna BazanMobayode AkinsoluAnna KarczemskaMohamad Ali BijarchiAnna Knitter-PiątkowskaMohamad HeriawanAnnalisa VolpeMohamadali MalakoutianAnnie KumarMohamadreza AfrasiabiAnthony BoydMohamed Al KhalfiouiAnthony CoustouMohamed Gamal MohamedAnthony J. ClarkMohamed HssikouAnthony Shun Fung ChiuMohamed Meselhy EltoukhyAnton AntonovMohamed MoustafaAnton DianovMohamed S. Mohy-EldinAnton SdobnovMohamed Sh Abdel-WahabAnton V. NaumovMohamed SharafeldinAntonella RosiMohammad Abdul AlimAntonina KarlinaMohammad AlibakhshikenariAntonino GulinoMohammad AmeenAntonio Alessio LeonardiMohammad Asif ZamanAntonio BalenaMohammad GheibiAntonio BarlettaMohammad H. HasanAntonio Pio CatalanoMohammad Hazhir MozaffariAntonio RoqueMohammad Hossein MaghamiAntonio RubinoMohammad I. YounisAntonio Sergio SombraMohammad MalikanAntonios GasteratosMohammad Masum BillahAntonios MakridisMohammad Mosarof HossainAnuar De Jesus Fernandez OlveraMohammad RezaieAnupam AgrawalMohammad Robiul HossanAnurag ThantharateMohammad SaadatiAnushka GuptaMohammad Saeid GhaffarianAnushree DuttaMohammad Tavakkoli YarakiAnvesh GaddamMohammad Yaghoub Abdollahzadeh JamalabadiAnwar SaeedMohammad ZamanAnyang WangMohammad Zhian AsadzadehAnže SitarMohammadmehdi Shahzamanian SichaniAnzhu GaoMohammed A. AlmeshaalAo LiMohammed Altaf AhmedApostolos KorlosMohammed BourhiaApurba MahapatraMohammed Faeik Ruzaij Al-OkbyApurba RayMohammed M. HameedAraa Mebdir HoliMohammed Nazibul HasanAranyak ChakravartyMohammed RasheedArash AhmadivandMohandoss SonaimuthuArash Yazdanpanah-GoharriziMohd AhmadArchibong Eso ArchibongMohd Fairus Mohd YusoffAres Argelia Gomez GallegosMohd FarhanArfan GhaniMohd Shahir Bin KasimArif UllahMohd Zahid AnsariArifin Budiman NugrahaMohit BajajArindam PaulMohit Naresh ShivdasaniAritra BiswasMohssin AoutoulArjun Prasad TiwariMojtaba JoodakiArmando Francesco BorghesaniMonika BłaszczyszynArnaldo César PereiraMonika SaumerArnaud BertschMonika TrojanováArnaud DuchosalMoon-Seok KimArshad AfzalMorgan HamonArtem E. ShitikovMorteza ArameshArtem KozlovskiyMorteza BayarehArtem MarchenkovMorteza MohammadzaheriArtur AndruszkiewiczMostafa AzimzadehArtur HermansMostafa BehtoueiArtyom Dmitrievich ObukhovMostafa TaghizadehArun ButreddyMostafa ZaydanArun Kishor JoharMotohiro FujiyoshiArun Prasanth NagalingamMounika KodaliArun ThirumuruganMu Wen ChuanArunas StirkeMu ZhouArvind Kumar ShuklaMu-chun WangAsaad Kareem Edaan Al-mashaalMudaser UllahAshiqur RahamanMuhamad Kamil YaakobAshish SrivastavaMuhamad Ramdzan BuyongAshok ChhetryMuhamad Risqi Utama SaputraAshrafizadeh Seyed NezameddinMuhammad AamirAshwani KumarMuhammad AfzalAsma′ Abu-SamahMuhammad Ali ButtAswin Kumar AnbalaganMuhammad Ali SharAtchudan RajiMuhammad Asif Zahoor RajaAthanasios G. PapathanasiouMuhammad AtifAthith KrishnaMuhammad AzamAtilgan AtilganMuhammad Hafiz HassanAugustine O. NwajanaMuhammad Idrees AfridiAurelian MarcuMuhammad Mubasher SaleemAurélio Luiz Magalhães CoelhoMuhammad Muqeet RehmanAurelio SomàMuhammad Nasir MuradAvik SamantaMuhammad NisarAwadesh Kumar MallikMuhammad Salman Al FarisiAwais BokhariMuhammad Sohail ZafarAydi AbdelkarimMuhammad SulamanAyhan BozkurtMuhammad TahirAyhan CelikMuhammad TanveerAyman AlneamyMuhammad Umair KhanAzam AbdollahiMuhammad UsmanAzana Hafizah Mohd AmanMuhammad Waseem AshrafAzhar IlyasMuhammad Yasar RazzaqAziz AmineMuhammad ZadaAziz BachaMuhammad ZahidAzremi Abdullah Al-HadiMuhammad ZubairB. C. PrasannakumaraMuhannad AlmutiryB. NagarajaganeshMuharrem KaraaslanBabak MosavatiMuhib RahmanBadariah BaisMuhibUr RahmanBadreddine AyadiMukesh KumarBahareh Ghane MotlaghMukesh Kumar ThakurBaijie QiaoMukul SonkerBaitang JinMullaicharam BhupathyraajBaiwen LuoMuneeb IrshadBalasankar AthinarayananMurat Kaya YapiciBaolin WangMurugapandiyan PanneerselvamBapun BarikMusawenkhosi MkhatshwaBarbara BrunettiMustafa GünayBarbara GierobaMustafa Hikmet Bilgehan UcarBarbara WłodarczykMustafa KuntogluBarbaros CetinMustafa TurkyilmazogluBartłomiej AmbrożkiewiczMustaqeem MustaqeemBartłomiej GardaMuthaiah AnnalakshmiBaskaran Stephen InbarajMuthusankar EswaranBavencoffe MaximeMutsumi KimuraBayrakdar Muhammed EnesMy HedhammarBeatriz Garcia-BañosMyounggon KangBehrmann OleMyunghoi KimBenjamin CerjanN. Rajesh Jesudoss HynesBenjamin H. WunschNa ZhouBenoît IgneNabeel AbedBen-Ran FuNachappa GopalsamiBerend van MeerNadanai LaohakunakornBerenice Fernández-RojasNadejda HorchidanBerna Özkale EdelmannNadia BoutabbaBettina HeiseNadish AnandBhanu ShresthaNadya Vasileva DenchevaBharath Babu NunnaNag AnindyaBhishma PanditNagaraj NandihalliBhupendra PrajapatiNagaraj P. ShettiBian TianNagulu AravindBiao TangNaheem Olakunle AdesinaBiao ZhaoNahuel OlaizBilal H. AlsalamNajm M. Al-HosinyBilal JavedNallappan GunasekaranBin HuangNamdev Shankar HaraleBin LüNamrata Dewan SoniBin XieNan WangBin ZhangNan WuBinbin JiaoNandalur Ameer AhamadBinbin YingNandkishor M. DhawaleBing WangNanqi LiuBinghao WangNaoto ShirahataBingjie DangNaoufal El HachlafiBingpu ZhouNaramgari SandeepBingxi XiangNarendar VadthiyaBiplob BrmanNaresh Kumar DarimireddyBivas PanigrahiNaresh Kumar ManiBjörn KemperNareshKumar DarimireddyBlanca Lapizco EncinasNaseem AbbasBo LiangNasim ChitsazBo WuNatalia BourguignonBo ZhengNatalia Do Carmo CarvalhoBo ZhongNatalia KizilovaBogdan Cătălin ȘerbanNatalia PorotnikovaBogdan Stefan VasileNataliya SakharovaBohdan HejnaNataliya V. KazantsevaBoming YuNataša SamardžićBoqiang ShenNathália Beretta TomazioBoris N. KhlebtsovNathan CraneBosko NikolicNathan JacksonBoualem DjezzarNathan JeongBranko MiloradovicNathan LazarusBrian BirchNaveen KumarBrian D. PlouffeNaveen Kumar RBrian IkedaNavid KashaninejadBrian VohnsenNavid RabieeBrice SorliNavid SohrabiBrigitte BruijnsNazli FarajzadehBriliant A. PrabowoNeda LatifiBriliant Adhi PrabowoNeelam SrivastavaBrînduşa Alina PetreNeeraj GuptaBrita OlsonNehad Ali ShahBrojeshwar BhowmickNeil CollingsBrunella CorradoNeirivaldo Cavalcante Da SilvaBuddhadev PurohitNektarios KoukourakisBudimir MijovićNerija ZurauskieneBülent DelibasNestor Méndez-LozanoBurak EminogluNghia HuynhBurhanettin KocNgoc NguyenBushra JabarNguyen Thuy ChinhByeong Yong KongNguyen Truong ThinhByeong-Ui MoonNguyen Van AnhByung Chul JangNguyen Van HoaByung Jun JungNi Nyoman KencanawatiByungil HwangNiamat HussainCadmus Ann YuanNiandong JiaoCalvin EiberNiaz Bahadur KhanCamelia UngureanuNiazul Islam KhanCamila D. Madeira CamposNibaldo RodriguezCamilla KärnfeltNicholas Scott Lynn Jr.Can HuangNico SuranthaCan WangNicola LiberatoreCan WuNicolae PopCao TanNicolás Laguarda-MiróCarla OrgeNicolas PerezCarl-Mikael ZetterlingNicole Y. MorganCarlos Alberto Ferreira MarquesNicolò LagoCarlos David Grande TovarNidhal HnaienCarlos FrajucaNidzamuddin Md. YusofCarlos HernandezNikhil BhallaCarlos Manuel Alves Da SilvaNikhil TiwaleCarlos MarquesNiklas WolffCarlos SabínNikola SakačCarlota Ruiz De GalarretaNikolai TsvetkovCarmela Tania PronteraNikolaos ChamakosCarmen MannellaNikolaos E. KarkalosCarmine CiofiNikolaos G. SemaltianosCatalina-Alice BrandusNikolaos TapoglouCaterina CrediNikolay DolgovCaterina GaudiusoNikolay DoynovCatherine MarichyNikolay PolyakovCederick LandryNikolay SmaginCelso A. R. L. BrennandNikolina JankovicCenk CelikNil PandeyCésar Eduardo Damián-AscencioNilankush AcharyaCeyhun KarpuzNilesh GoelCh Rami ReddyNileshi SarafChaitanya MahajanNimal Thattaruparambil RaveendranChandan JoishiNina FincurChandan PandeyNina KravetsChandra B. KhatriNino F. LäubliChandrakant Kumar NiralaNino LäubliChandreswar MahataNitesh NamaChang Gyu WooNives StrkaljChang LiuNoah SternChang PengNobuyuki FutaiChanghyuk LeeNoel PereraChang-Seuk LeeNomin-Erdene OyunbaatarChangsheng LiNoor Asmawati Samsuri Noor AsmawatiChang-Wei ChengNoorina Hidayu JamilChang-Whan LeeNor Bahiyah BabaChangyong GaoNoriyuki KawarazakiChangyong YimNosherwan ShoaibChangzhou YuanNupur NavlakhaChao ChangNur Azura Mohd SaidChao LuNurettin SahinerChao MengNúria BarniolChao TanNuttapol TanadchangsaengChao WangNwagoum Tuwa Peguy RousselChao ZengOana DodunChao ZhenOcak Yusuf SelimChaobiao ZhouOday Ibraheem AbdullahChaofu WangOh Seok KwonChao-Hsin WuOk Chan JeongChao-Hui FengOksana KomovaChaojiang LiOlajumoke OyebodeChaomin ZhangOlcay AltintasChaowei SiOle HölckChen HuOleg A. StepanovChen LiOleg LeonovChen XiaoOleg VitrikCheng Ee MengOlga BoytsovaCheng GuoOlga DruzhinaCheng HuangOlga SamoilovaCheng WangOliver HöfftCheng ZhangOliver OziokoChengfa WangOltmann RiemerCheng-Hsien LiuOluwatosin OluwadareCheng-Hsun-Tony ChangOmar ElmazriaCheng-Huan ChenOmar Faruk EmonChengkuo LeeOmid MahdavipourChengming JiangOmneya AttallahCheng-Mu TsaiOn Shun PakChengning WangOnder ErinChengtao YuOpeyemi A. OsanaiyeCheng-Yang LiuOPhir NaveChenxi YangOrchidea Maria LecianChenyuan HuOsamu KamiyaChia-Chen ChangOscar CastilloChia-Jung ChoOsman UlkirChia-Min YangOualid DjekouneChia-Ming ChangP. M. Durai Raj VincentChiao-Chi LinP. N. AnantharamaiahChiara FedeleP. PoorneshChia-Yen HuangP. V. Satya NarayanaChiayi HuangP. Vigneshwara RajaChi-Ching ChangPablo A. KlerChien-Nan KuoPablo Giménez-GómezChih-Cheng HuangPablo Luis López EspíChih-Lang LinPablo Palacios-JátivaChih-Liang WangPalamandadige K. S. C. FernandoChing-Chi HsuPalaniyappan SabarinathanChing-Hwa ChengPalilis LeonidasChi-Vinh NgoPalla GopalChizhao YangPallavi GuptaChong LiPanagiotis Karmiris-ObratańskiChongjing CaoPandiyarasan VeluswamyChris BadenhorstPaola Di MatteoChris O. PhillipsPaola Di PietroChristabel TanPaola Serena GinestraChristian A. ZormanPaolo MilaniChristian BretthauerPaolo SgarbossaChristian Chapa GonzálezPaolo TrucilloChristian GaleaParamsamy Kannan VimalathithanChristian GaussParvez Ahmad AlviChristian MaibohmPascal NicolayChristian ThomPathak SunilChristine WünschePatrice MélinonChristoph FaschingerPatrick FaircloughChristopher L. FrewinPatrick PonsChristopher OshmanPatrik RohnerChristos D. NikolopoulosPaul C. GowChristos MichailPaul G. RoyallChristos NikolopoulosPaul MotzkiChristos S. AntonopoulosPaul TucanChuan LiuPaulina ChilimoniukChuanbing ZhangPaulius SakalasChuang QuPaulo CaldasChuang ZhuPaulo GoncalvesChuanyin XiongPaulo M. MendesChuanyu SunPaulo SantosChuen-Lin TienPaulo Sérgio De P. Herrmann JúniorChul Wan ParkPavan ChaturvediChun CaoPavan PujarChun ZhaoPavel ChulkinChun-Chieh ChangPavel DemakovChundong XuePavel GrigorievChung Chen TsaoPavel H. Lugo-FabresChung-chih WuPavel HazdraChung-Wei ChengPavel Ivanoff ReyesChun-Ho LinPavel KhramtsovChun-Hsiung LinPavel LafataChunjie ChenPavel LaryushkinChunjin WangPavel NosovChun-Liang YehPavel ŠkarvadaChun-Ping JenPavel TuralchukChunya WangPavol KurdelChun-Yen LiuPaweł GóreckiChunyi SuPaweł LigęzaChunYing HuangPawel RzucidloCian McCaffertyPaweł SułkowiczCihun-Siyong (Alex) GongPaweł TurekCindy BerriePawel ZylkaCinzia CaliendoPay Jun LiewCiprian IliescuPedro Andre PratesCiprian NemesPedro Bertemes-FilhoCiro ScognamilloPedro CruzCk SumeshPedro FaiaClarice SteffensPedro FernandesClassius Ferreira Da SilvaPedro Vargas ChabléClaude H. YoderPei Ling LeowClaudia CampanalePeihua YangClaudia WeidlichPeilong HouClaudio TestaniPeiman GhasemiClemens K. WeissPeiran ZhangCongwei WangPeiyan WangCorey ShemelyaPeng LiangCornel CobianuPeng ShaoCornel SamoilaPeng SunCristian PetcuPeng WangCristiane KalinkePeng ZhangCristiano FragassaPengcheng ZhangCristiano PalegoPengfei LuCristina PotrichPengfei ZhangCristina RusuPeshala GamageCui PengPetar PetrovicCunlu ZhaoPeter HeskethCunteng WangPeter KoledaCynthia HajalPeter KorbaCzang-Ho LeePeter LukácsD. Arul KirubakaranPeter ScharffDaeseok LeePeter ŠugárDaewon KimPeter VerstraelenDagmar GregušováPetr BoriskovDalei JingPetr FilipDalili ArashPetr JaníčekDamir KapetanovićPetr KudrnaDan ButnicuPhan Huu NguyenDan DobrotaPhilip TroykDan WuPhilippe ChristolDan YuanPhilippe VelhaDana Elena PopaPhilippos PapaphilippouDana Mihaela CristeaPhillip WonDandan XiaPierre ChavelDaniel Basso FerreiraPierre-Alain FayolleDaniel BenedikovicPi-Gang LuanDaniel BudaszewskiPilar ViannaDaniel CarlPinar Aydogan GokturkDaniel CruzePing YuDaniel HeadlandPingan ZhuDaniel Hernandez-BalbuenaPingkeng WuDaniel HoffmannPiotr GarbaczDaniel Jáuregui-VázquezPiotr GębaraDaniel McGrathPiotr GorniakDaniel RamosPiotr KuwalekDaniel TomaszewskiPiotr M. MarkowskiDaniela DragomanPiotr MrozekDaniel-Constantin AnghelPiotr SleczkowskiDaniele CafollaPiotr WisniewskiDaniele Eugenio LucchettaPiotr WróblewskiDaniele PincheraPiyush GuptaDaniele Ugo TariPlácido Rogerio PinheiroDaniele VellaPo An ChenDanil Yu. LyapunovPolina A. DeminaDanny ReuterPolina P. KuzhirDanqing WangPoonam SundriyalDapeng WangPoongavanam GaneshKumarDaquan YuPooria GolvariDardan KlimentaPouya BarnoonDariusz GrzybekPo-Wen ChenDariusz KlepackiPrabal BasuDariusz LitwinPrabhakar BhandariDariusz MikielewiczPrabhakar SinghDariusz TomkiewiczPrabhat KumarDarjan PodbevsekPrabhu SethuramalingamDarya TishkevichPrabir HaldarDasaroju GangacharyuluPrabu SeetharamanDastjerdi ShahriarPradip DasDavid CameronPragya SinghDavid FolioPrahar M. BhattDavid G. NairnPrakash Chandra MishraDavid GrassPrakash JayavelDavid GuiraudPrakash PitchappaDavid JenkinsPramod K. KalambateDavid KumarPranab Kumar MondalDavid ValientePranav BhounsuleDavid WamplerPrasenjit MondalDavide FerraroPrashant KumarDavide MasatoPrateek BenhalDavor VinkoPratik M. PataniyaDawei LiangPraveen ChoppalaDawei ZhaoPraveen K. JainDe GongPraveen KumarDe Xing LioePredrag DašićDebadatta SethyPredrag KojicDebajyoti BhaduriPredrag PetrovićDebarun SenguptaPrince Waqas KhanDebashis MajiProloy Taran DasDebasis MaityPrzemyslaw LedwonDebkalpa GoswamiPrzemyslaw SowinskiDebsindhu BhowmikPulak BhushanDecha DechtriratPunam MurkuteDeepak KulkarniPyo-Woong SonDeepak Kumar PokkallaQasem M. Al-MdallalDeepak SharmaQi ShaoDehui WangQi WangDejan BrkićQian WuDejan V. TosicQian ZhangDejiu FanQianbin ZhaoDelly RamadonQiang GaoDenis BalzamovQiang LaiDenis KarimovQiang LiuDenise BellisarioQiang ZhangDesalegn T. DebuQiang ZhouDesu ChenQianqian LiDetlev MarkusQicheng ZhangDhanoj GuptaQifan LiDharamdeep JainQiguang HeDharmendar Reddy YanalaQiji ZeDheeraj K. SinghQijun LuDhésmon LimaQilin HuaDhiman DasQilong ZhaoDhruvajyoti RoyQimin ShiDi ZhangQing HaoDiana ChioibasuQing LuoDiana PinhoQingdong OuDiana VellutoQinglei GuoDianpeng QiQinglin LiDibakor BoruahQingwei LiaoDiego CarouQingyang DuDietrich KohlheyerQingzhen YangDilip Kumar AgarwalQinlin CaiDimitra N. PapadimitriouQiu XuDimitrios G. FatourosQiubo ZhangDimitris A. ChalkiasQiuhua GaoDimka I. IvanovaQiyao HuangDinesh PathakQu ZhouDinesh SabarirajanQuan DongDinesh Veeran PonnuveluQuanbin ZhaoDing NingQuang Minh NgoDingding RenQuansan YangDingnan DengR. J. PunithgowdaDinh Gia NinhR. N. PonnalaguDinu DraganRachel A. SaylorDiogo E. AguiamRachid SaadaneDipankar MitraRadosław NowakDirk RueterRadovan MetelkaDivij BhatiaRadu-Eugen BreazDjordje VukelicRaees SwatiDmitrii Andreevich SinevRafael Vargas-BernalDmitrii IrzhakRafał BielasDmitriy N. ArtemyevRafał GołębskiDmitriy Yu. OzherelkovRafal KowerdziejDmitry AldakovRafal PrzesmyckiDmitry L. ZaitsevRaffaele GiordanoDmitry LevshunRafiqul I. NooraniDmitry MarkovRagwa FaridDmitry MogilevtsevRahim AbdurDo Jin ImRahul Kumar JaiswalDobrochna Ginter-KramarczykRahul MishraDoina FrunzaverdeRahul MulikDomin KohRaimonds MeijaDominic J. WalesRajan Kumar SinghDominik WyszyńskiRajanand Patnaik NarasipuramDominique PagnouxRajaprakash RamachandramoorthyDomna G. KotsifakiRajat M. MahapatraDong HanRajender BoddulaDong Ho LeeRajesh Kumar TripathyDong Hoon LeeRajesh SinghDong JiRajkishor KumarDong WangRajkumar SarmaDong WuRakesh ChaudhariDong YeRakesh Nath TiwariDong Yun ChoiRaktim HalderDongdong HanRalf BauerDongfang LIRalph HolzelDongfeng HeRam Kumar KarshDongin KimRamachanran KasuDong-joo KimRamesh MandaDonglai ZhongRamón González RubioDonglin ZouRamón Moreno-TostDongling ZhouRan ChenDongshi ZhangRasmi Ranjan BeheraDong-Wook SeoRastislav RokaDong-Yeon LeeRatheesh Kumar MeleppatDoo-Seung UmRathinam BalamuruganDorian CojocaruRaul Borja UrbyDorijan RadočajRaúl GagoDorit Esther ZilbermanRaul RabinoviciDorota StachowiakRaul-Cristian RomanDouglas Vieira ThomazRavi KadlimattiDragos AmarieRaviraj ThakurDuanzhi DuanRazvan Ioan PacurarDuc-Nam NguyenRebeca De NaldaDumitrescu CatalinRebeca Martínez VázquezDun-Bao RuanRebeka RudolfDung-An WangReihaneh JamshidiE. V. KunitsynaRemberto Sandoval-ArechigaEbrahimi MehdiRemigijus JuskenasEce UykurRenan TrevisoliEdgars ElstsRenata Kelly MendesEdiguer Enrique FrancoRenata SzydlakEdoardo SinibaldiRevannath Dnyandeo NikamEdson SantanaRex Xiao TanEduard BorrasReza GhiaasiaanEdward BormashenkoReza KhayatEdwin En-Te HwuReza NorooziEdzrol Niza MohamadReza RoohiEfrain Mejia-BeltranRhokyun KwakEhsan RoohiRicardo M. F. FernandesEilon FaranRicardo OliveiraEirini Eleni TsiropoulouRiccardo ZamboniEirini MyrovaliRicha SharmaEjay NsugbeRichard G. ForbesEjaz HussainRichard HoodEkaterina PavlyuchenkoRichard HopperElaine LeeRichard MauliniEldad Bahat-TreidelRidha HorchaniElena AnashkinaRika OchiElena HelereaRiko ŠafaričElena N. PivovarovaRio Prasetyo LukodonoElena SalernitanoRishibrind Kumar UpadhyayElena SolovyevaRita GraceffaEleonora AlfinitoRita KadikovaElisa CairrãoRitamay BhuniaElisa FracchiaRitesh Kant GuptaElisabeth GillRitesh Ray ChaudhuriElisabetta Di FranciaRitu RavalElisabetta MoiselloRoald M. TiggelaarElizaveta EvshchikRobert CzarnotaEl-Sayed ShalaanRobert KosturekEluri PavitraRobert MingeszElvira RozhinaRobert NusterEmil CazacuRobert PolasikEmil SmykRobert PrzekopEmile MartincicRoberto Alves BragaEmiliano Martínez-PeriñánRoberto Baginski B. SantosEmiliano ZampettiRoberto Bernal-CorreaEmilio AndreozziRoberto Maria-HormigosEmma Panzi MukhokosiRocco CancelliereEmmanouil-Lazaros PapazoglouRocco CrescenziEmmanuel Karlo NyarkoRocío CánovasEnache StanicaRodica VoicuEngin CiftyürekRodrigo PicosEnrico VeronaRoer Eka PawinantoEnwei SunRoger C. LoEnyi YaoRohit SharmaErcan KoseRohith MittapallyErcan YilmazRohollah NasiriErdenebileg BatbaatarRoland RyndzionekErdong WangRolandas TomasiunasEric BideauxRoman GrillEric ChappelRoman MykhailyshynEric FujiwaraRoman SavraiEric LeeRoman StoklasEric StarkeRomana SchirhaglEros Alexandru PatroiRomolo MarcelliErsin KayahanRonaldo CozzaEryk WolarzRong XiangEsam YahyaRonghuai QiEshwar ThoutiRonghui WuEsmaeil DehdashtiRoozbeh Abedini NassabEssia HannachiRoozbeh TabrizianEsteban Sánchez HernándezRory WatermanEsteban Tlelo-CuautleRosa Ana Perez HerreraEsteve Amat BertranRosminazuin Ab. RahimEuclides Lourenço ChumaRossen TodorovEugene A. AvrutinRoungsan ChaisricharoenEugenijus GaizauskasRoy KnechtelEun Kwang LeeRoy PembertonEunho LeeRoyson Donate DsouzaEusebio BernabeuRuben ForestiEvangelia TzanetouRubina ShaikhEvangelos KarvelasRucha NatuEvelio Ramirez MiquetRuchir PriyadarshiEvgenia BenovaRuei-Sung YuEvgeny BuntovRuey-Ching TwuEwa Juszyńska-GałązkaRufat AbievEyad HamadRugved LikhiteEzequiel VidalRui A. LimaFabao YanRui ChengFabien GriséRui Jorge Coelho GusmãoFabio Di NardoRui MinFabio ImmovilliRui Pedro MartinhoFabio SimõesRui ZhaoFabrice SthalRuifang LiuFabrizio MatteiRuifeng LiFadi AlsaleemRuiheng LiuFaezeh ShanehsazzadehRuihua DongFahad AlamRuimin ChenFaiz ArithRuinian JiangFaizan QamarRuining HuangFan YangRuisong WangFang WangRuitao YangFang YuanRujiang LiFanny MousseauRunchen FangFarhad Rezai-AriaRunchen ZhaoFarhad SattariRunsheng XuFarid ZubirRuoshi ZhangFaridah SonsudinRussell FarrugiaFarkhondeh JabariRyan RudyFaruk DirisaglikRytis ButkusFaruk Fonthal RicoS. AlwinFaruk ÖzgerS. NallusamyFateh Mebarek OudinaS. ValkovFatema H. RajabS.S. VermaFatemeh Ghorbani-BidkorpehSaad Hassan KianiFatma Savran OguzSaadi BerriFayçal DjeffalSaba MosivandFazal MuhammadSabapathy ThennarasanFederico MoroSabina BottiFedor SmirnovSabrina RoguaiFei LiuSachin KumarFei WangSachin SharmaFei XingSachin SirohiFei YangSaeed Ahmed KhanFei YuSaeed DinarvandFeichi ZhouSaeed OlyaeeFelice GesueleSaeed RoshaniFelicia ToleaSagar M. DoshiFelipe Garcia-SanchezSagar ManeFen XueSagnik BasurayFeng ChenSai ChenFeng GuoSai Kiran OrugantiFeng LiSaid BenkhayaFeng LiuSaikat MaitiFeng WangSaikat Ranjan MaityFeng ZhaoSajad Abolpour MoshiziFengman LiuSajal Sagar SinghFeng-Tso ChienSajid Ali AnsariFerdows AfghahSakellaris MailisFerenc Tolvaly RoșcaSaket KaushalFernandes Pinto InêsSalah I. YahyaFernando De Souza CamposSalam KhamasFernando Henrique CincottoSalil BapatFernando Lessa TofoliSalman AkhtarFernando M. Araujo-MoreiraSalman AliFernando Martinez PedreroSalman ArainFernando Verdú Vidal-VerdúSalmon LandiFesenko HermanSalvatore SurdoFikret YildizSam KimFilip GucmannSam PeringFilipe AlvesSamaneh Salkhi KhasraghiFilippo PederzoliSamantha HarveyFirman Mangasa SimanjuntakSamara FirebaughFleming GudaguntiSambandam AnandanFlorina TeodorescuSameer JoshiFoad GhasemiSami FranssilaFoo Wah LowSami MyllymaaFrances AllenSampath BoopathiFrancesca CirisanoSamuel Kofi ErskineFrancesca LeonardiSamuel M. SilvestreFrancesco Alessio DicandiaSanatan DasFrancesco CacciolaSandeep K. ChaudhuriFrancesco CostanzoSandi Baressi ŠegotaFrancesco FerranteSandip DuttaFrancesco FusoSandip SarkarFrancesco Giuseppe Della CorteSandra PralgauskaitėFrancesco GrecoSandro LongoFrancesco Marotti De SciarraSang Hoon OhFrancine KriefSangeeta SinghFrancis LinSang-Hoon BaeFrancisca SeguraSangjin ByunFrancisco AznarSangsoon LimFrancisco Javier MedelSangtae AhnFrancisco Jiménez-MolinosSang-Woong YoonFrancisco PasadasSanja ErakovićFrancisco PizarroSanjaya BandaraFrancisco SilvaSanjeev Shashi KumarFrancisco Tovar LopezSantan KumarFranck BadetsSaqer AljaafrehFrancois Le NormandSarah De MarchiFrancois MoriniSarris IoannisFrancois RaultSasa SladicFrank FournelSasha Cai Lesher-PérezFrank P. MissellSathish DharaniFrank R. IhmigSathish ThanikodiFrantišek KlimendaSathiyaseelan AnbazhaganFrantisek UherekSatish KumarFranz L. DickertSatoshi NinomiyaFrédéric DumurSatyendra Kumar MishraFrédérique DucroquetSaulius JuodkazisFugui XieSawsan Khader DagherG. C. ShitSayak DasguptaGábor GyarmatiSayed Ali Ahmad AlemGabor KosaSchmidl GabrieleGabriel Luna-BarcenasSebastian EnglartGabriel NallathambiSebastian HenselGabriela Figueroa MirandaSebastian Marian ZahariaGabriela Iulia DavidSebastian SchwamingerGabriela Valdés-RamírezSébastien HarleppGabriele NavaSedat OzerGabriele PapadiaSeigo OhnoGaddale Amba Prasad RaoSeiji FukushimaGaetano D. GargiuloSelim GurgenGaia MaselliSelvam ChandrasekaranGaia RocchittaSenghane MbodjiGaitan Nicoleta CristinaSenho SeokGalina A. KovalenkoSenthil Kumaran SelvarajGalina V. KurlyandskayaSenthilkumar MohanGang GeSeok Jae LeeGang YuanSeok-Ho RhiGaobo XuSeong Tak WooGaofeng ZhengSeong-Min LeeGaojian HuangSeonho SeokGaoming LiSerena De SantisGaozhe CaiSerge OstrovidovGarbas Anacleto Santos JuniorSergei KomogortsevGary Garcia-MolinaSergei KulinichGaspare GiovincoSergei RyzhkovGaulthier RydzekSergej OrlovGautam MahajanSergey A. KukushkinGayathri PillaiSergey FedotovGeetesh K. MishraSergey GaponenkoGelu GurguiatuSergey I. KudryashovGemma García MandayoSergey KobtsevGeng-Bo WuSergey KrasikovGengxi LuSergey RusakovGeon Hwee KimSergey V. DorozhkinGeorge KaralisSergi GallegoGeorge MelillosSergii ChertopalovGeorgi Gary RozenmanSergio A. DavidGeorgia D. KaprouSergio SpanòGerald UrbanSergiu IordanescuGérard Liger-BelairSergiusz ŁuczakGergana G. NestorovaSergiusz PatelaGerhard FranzSerigo AcostaGerhard MullerSeunghwan BaeGerman Augusto Ramirez ArroyaveSeungil KimGevork Ya KarapetyanSeung-Jae MoonGéza István MárkSeungwon JungGhazal A. FahmySevinc KurbanogluGheorge IliaSeyed Mohammad RazaviGheorghe NagitSeyed Mohsen SerajiGheorghe PristavuSeyed Rasoul AghazadehGhosh DebanjanaSeyed Reza Mousavi AghdamGhulam DastgeerSeyed Soheil Mousavi AjarostaghiGhulam HafeezSeyedfakhreddin NabaviGiacinto BarresiSeyed-Sajad AhmadpourGianandrea PasquinelliSeyyed Mojtaba MousaviGiancarla AlbertiSh D. PayziyevGiane G. LenziShaahin AngiziGianluca GattiShady MagedGianni BosiShahab GolshanGiedrius JanusasShahadat Hasan SohelGilberto Anzueto-SánchezShahidul Islam BhatGilliard SilveiraShahrokh HatefiGina AmbrosioShaloam DasariGintautas BagdziunasShangquan WuGioele CapilloShankar Goud BejawadaGiorgio PennazzaShankar SubramaniamGiorgos DimitrakopoulosShanshan LiGiovanni CeccioShanti BhattacharyaGiovanni CrupiShaohong ShiGiovanni Di LibertoShaohua PiGiovanni GhioneShaojian ZhangGiovanni GugliandoloShaolong WuGiovanni VitaleShaoze YanGirolamo CostanzaSharafaldin Al-MusawiGiulia ButtazzoniSharat Chandra BarmanGiuseppe ArteseShashi PrakashGiuseppe BarillaroShau-Chun WangGiuseppe BrunettiShawn SanctisGiuseppe CastaldiShazia HasanGiuseppe Di MassaShengchuan WuGiuseppe FerriSheng-Hsiang TsengGiuseppe MaruccioShenglin MaGiuseppe PesceShengzhou BaiGiuseppe StaraceShi ChenGiuseppina GiniShiba DandpatGiwan YoonShibiao WanGleb M. KatybaShibnath SamantaGleb TselikovShibo ChengGlenn RossShih-Hung LinGobinath JegannathanShih-Jeh WuGobinath RavindranShih-Kun LiuGolam KibriaShimba KentaGomaa El FawalShinpei OgawaGonçalo TiagoShiny Amala Priya RajanGong ZhangShipin QinGongchen SunShirin NasresfahaniGongye ZhangShiromani Balmukund RahiGonzalo IriarteShirshendu DasGooi Mee ChenShishang GuoGopala Krishna PodagatlapalliShital S. ChiddarwarGopi Chandra AdhikariShivani SathishGorka UrbikainShivatharsiny RasalingamGour Mohan DasShiyu YueGourab MajumdarShiyuan LiuGovindasamy ManiShouhu XuanGovindhan MaduraiveeranShoyebmohamad F. ShaikhGregory ChassShrish PatelGrigory V. ZyryanovShruti NirantarGrzegorz BazydłoShuai LiGrzegorz BlakiewiczShuaishuai SunGrzegorz JanczykShuang ZhangGrzegorz KrólczykShuangyang ZouGrzegorz MieczkowskiShubhendra JainGrzegorz SkrabalakShubhodeep GoswamiGrzegorz SobczakShubhra S. PasayatGrzegorz StruzikiewiczShude LiuGrzegorz ZielińskiShudhashil BharthuarGuangfu WuShuji TanakaGuangtao ZanShujie YangGueorgui Kostov GueorguievShulin ChenGufran S. KhanShunbo LiGuijian XiaoShunping ZhangGuilherme B. LucasShuntaro TsubakiGui-Lin SheShunyu LiuGuillermo Asín PrietoShuo SuiGuillermo Garcia ToralesShuo YangGuimin ChenShuting LeiGulsim KulsharovaShuxiang GuoGunawan Setia PrihandanaShuye ZhangGunel HuseynovaSiamak GhorbaniGunnar SuchaneckSiavash AhrarGuo JiangSicong SunGuodong ChenSid Ahmed SlimaneGuodong ZhouSiddhartha TripathiGuo-En ChangSifani ZavahirGuolin YunSikandar I. MullaGuorui JinSilvia RizzatoGuoxu LiuSilviu Dan MandruGuoying DongSima NoghanianGurpreet SinghSimeone DussoniGurumaddaiah Ajay KumarSimon T. AbramsGururaj BolarSimón Yobanny Reyes-LópezGururaj Kudur JayaprakashSimona NoveanuGustavo Foresto Brito AlmeidaSimone PiacentiniGyudo LeeSimranjit SinghGyula GrófSinan GucluerHabib HamamSiniša BikićHacı SaglamSiva Kumar ReddyHadi HashemzadehSivakumar RajamanickamHadi MirzajaniSiyuan HeHaedong ParkSlawomir PietrowiczHae-Jin KimSlobodan MoračaHai LiSlobodan RadojevicHaibo GongSneha SamalHaibo ZhangSnehashis PalHaichao WuSnezhana HristovaHaiding SunSobhan RoshaniHaidong FengSofia CeccarelliHaifei ZhanSofia JohanssonHaifeng XuSofia MorozovaHaihui Joy JiangSohel RanaHaipeng LiSohichi HiroseHairong LinSojung KimHaisong LinSomyot ChirasatitsinHaitao LuoSong GaoHaiyang MaoSong YangHaiyi LiangSongbai ZhangHakan F. ÖztopSonglin ZhangHakan KuntmanSonglin ZhuangHamad SyedSongwang YangHamdi Ben HalimaSongyuan ZhangHamed Aghajani DerazkolaSonia RobertsHamed NosratiSoo-Ho JoHamza ShakeelSook Mei KhorHan Eol LeeSophia Ogechi EkeukuHan HuSoraya CaixeiroHan ZhangSorin Ioan DeaconuHangbo YangSorin Vasile SavuHangrui LiuSoshi IimuraHanif AdeelaSoumei BabaHanna V. BandarenkaSoumendu SinhaHanyang LiSoumya SrivastavaHao LiuSourabh GhoshHao LongSourangsu BanerjiHao SongSourav DeHao YuSrecko StopicHao ZhengSrete NikolovskiHaobo YuanSridhar ChandrasekaranHaochen LiSrinivas PadalaHaochong HuangSriram KesarimangalamHao-chung KuoStanislao GraziosoHaoran WenStanislav SazonkinHaotian ChenStanisław NogaHaotian ZhuStefan GassmannHaoyang ChenStefan KöstlerHaoyang CuiStefan RombachHari Prasad KamatamStefan SchröderHariharan NhalilȘtefan ȚăluHarish HandralStefan ZirnHarjit SinghStefania BonafoniHarold PhilipsenStefania MarsiliHaroon Ur RasheedStefania ZappiaHarsh ChaliyawalaStefano MarianiHarsha BajajStefano PelliHaruyuki SakuraiStefano ZampolliHassan AlgadiStela DimitrovaHassan Al-KaragolyStewart SmithHassen OuakadStoyan SlavovHayde Peregrina-BarretoSu Yun-HsuanHayrettin KöymenSubhajit ChakrabortyHaytham El-gazzarSubhajit RoychowdhuryHe DingSubhashish DolaiHeba A. GadSubhradeep PalHeba AhmedSubhrokoli GhoshHéctor GarcíaSubing QuHéctor García-MartínezSudeshna SamantaHeetae KimSudhansu DasHeiddy P. QuirozSudhansu Ranjan DasHelena FelgueirasSudhir ShendeHemanth GudapatiSudip Poddar‪Hengxu SongSudipta DasHenrique Leonel GomesSufang LiuHenrique M. SalgadoSuguo HuoHeon-Ho JeongSuhas KumarHesham MohamedSuhas S. JainHexiu XuSuhui LeeHeyong XuSujan ShresthaHicham Mahfoz KotbSujan YenugantiHijaz AhmadSujit PardeshiHilal ZaidSujoy Kumar GhoshHimanshu SharmaSukhveer SinghHimarati MondalSumanta SahooHiran LalSumanth Kumar PavuluriHiroaki KikuchiSumei HuangHiroshi MaiwaSumit Kumar JindalHirotada HiramaSunbin DengHitesh BorkarSuneetha VuppuHithesh Kumar GattySung Il AhnHitoshi MugurumaSung Sik LeeHoa Thanh LeSungchan YunHoàng Ly NguyễnSung-Eun ChoiHoe Joon KimSungho JeonHong DingSunghoon HurHongbao XinSungjun KimHonghao HouSungjun ParkHongjie HuSungjun YooHongliang LuoSungjune ParkHongmiao TianSung-Min KangHongnan XuSunhee LeeHong-Quan NguyenSunil PathakHongrui AoSuping ShenHong-Seok LeeSurasak KasetsirikulHongwei LeiSurbhi SharmaHongwei QuSurekha YadavHongwen ZhangSuresh NuthalapatiHongya LuSurjit SahooHongyang ZhuSurojit ChattopadhyayHooman YarmandSusan ZhouHorn-jiunn SheenSusana CatarinoHossain Md. BellalSushanta SethiHossain NematiSutirtha ChowdhuryHossein AminiSutripto MajumderHoupu LiSuyel NamasudraHouqiang FuSuzan MeijsHo-Young ChaSuzana UranHristos T. AnastassiuSvetislav SavovicHsiang-Chen ChuiSvetlana KonnovaHsiao-Mei WuSvetlana KotovaHsieh-Fu TsaiSvetlana NalimovaHsien-Tsung WuSviatlana MarozavaHsuan-Ling KaoSwami Vetha Berwin SinghHsueh-Chan LuSwee Leong SingHuabiao ZhangSyed Farid Uddin FarhadHuafeng LiuSyed Salman ShafqatHuaizhong LiSyeda Iffat NaqviHuamin ChenSy-Hann ChenHuamu XieSylvia GebhardtHuan TsaiSylwia BalutaHuan WangSzu-Hao ChenHuang XiaowenSzymon WojciechowskiHuanhuan FengT. Thang Vo-DoanHuanyu JinTadao HashimotoHugo José Nogueira Pedroza Dias MelloTadeusz MikolajczykHugo S. OliveiraTae-Hak LeeHui JingTaehui NaHui LiTaejin LeeHui LiuTaeyoon KimHuijun XieTaha A. ElwiHuili LiuTaher ArmaghaniHuilin ShangTakaharu YaguchiHülya öztürk DoğanTakashi MatsumaeHumberto Martínez-BarberáTakehiro TachizakiHumphrey AdunTakuo SakonHung NguyenTalha YaqubHung Tran NguyenTalib Al-AmeriHung-Chang LiaoTamal RoyHung-I LinTamara TomašegovićHunter RauchTamás HaideggerHuolin HuangTamoghna SahaHusain JafferyTanmoy PramanikHüseyin PolatTanveer AhmadHussein Mohammed Abdel Moneam HusseinTanveer FaridHutomo Suryo WasistoTanweer AliHuy Hung TranTao ChenHwang-Cherng ChowTao HuangHyeon-June KimTao LiHyeonseok KimTao WangHyeun Joong YoonTao WuHyo Jae YoonTao YangHyun KimTao YuanHyungseok ChoTao YueHyunseok KoTarek KapielI. V. NelasovTarek TahaIan FouldsTariq Ahmad SofiIbrahim MohamedTatiana PriamushkoIc Pyo HongTatsuro GodaIevgen ZaitsevTatsuya FunazukaIgnacio Enrique Zaldívar-HuertaTatsuya IwataIgor KorobiichukTayeb BrahimiIgor KostolnýTeng ChiIgor LitvinchevTengjiang HuIgor MerkuryevTerenziano RaparelliIgor PoperechnyTeresa BajorIhsan Ali GhaniTero TuovinenIjaz GulThang Phan NguyenIkram UllahThanh Luan PhanIkramullah KhosaThanh-Canh HuynhIldikó MóczárThar M. AlbarodyIlia RasskazovTheodorakos IoannisIlker BayerTheodoros KarakasidisIlya ZudinThiagarajan SoundappanIlyas KhurshidThiago SequinelImran AliThiago Valle FrançaImre BakonyiThilo SandnerInam UllahThittaporn GanokratanaaInam Ullah KhanThomas BlanchetIndrek MustThomas ChristopoulosIngo KampenThomas HenkelInyong MoonThomas LindnerIoan BurdaThomas M. KraftIoan UrsuThomas StaubliIoana DemetrescuThomas VasileiadisIoannis D. ChasiotisThomas WeiheIoannis P. GravasThomas WendtIoannis SamarasThuan Ngoc VoIonel ZaganThye Foo ChooIonica OncioiuTian ZhaoIonut Daniel GeoneaTiangui YouIosif-Vasile NemoianuTianlong LiIraklis AnagnostopoulosTianqi SongIrene Buj-CorralTianyu YanIreneusz KubiakTibor KrenickyIreneusz SzachogluchowiczTien NguyenIrfan BahiuddinTim W. C. BrownIrina BesliuTimur Sh. AtabaevIrina PapkovaTing HanIrina PetrushinaTing LiuIrina V. SemenovaTingfeng MaIruthayapandi Selestin RajaTingjin ChenIsa AnshoriTingting HaoIsaías E. GarduñoTingting YangIshak ErtugrulTitus SanduIsrael Martin-EscalonaTiziana RitaccoIsrat Rumana KabirToan Thanh DaoIssa Sulaiman Al-AmriTobias HemselIstvan RajtaTom SunnyIvalina AvramovaTom VerstratenIvan A. ParinovTomás Gómez Álvarez-ArenasIvan LimaTomasz BłachowiczIvan M. Suarez CastellanosTomasz DylIvan Miguel PiresTomasz KoziorIvan MrkvicaTomasz RozwadowskiIvan PelevinTomasz RymarczykIvan PuchadesTomasz StrachowskiIvana GadjanskiTomasz SzymborskiIvana PanžićTomasz UrbanowiczIvo BoblanTomasz WasilewskiIvo ButtinoniTomasz WejrzanowskiIvo StachivTomaž BrajlihIzabela PiegdońTomi T. LiIzabela ŚliwaToni BjörninenIzhar IzharToomas RangJ. AjayanToru ItagakiJ. G. ManjunathaToshiyuki DoiJaap Den ToonderToshiyuki MitsuiJacek AndrzejewskiTran VinhJacek NyczTrieu NguyenJacek SmerekaTristan Van LeeuwenJack MerrinTrushit UpadhyayaJacopo Maria De PontiTsuneo HoriguchiJae-Hyoung ParkTsung Hung LinJaejong LeeTsung-Yi HoJae-Min KimTsz Him ChowJae-Myung RyuTuan Anh PhamJagadheswaran RajendranTuan-Anh LeJagriti NarangTuo ShiJahangir MasudTze Chuen YapJaideep KaturiUdochukwu B. AkuruJaime J. HernandezUgur BozuyukJaime ViegasUlrich GengenbachJakub HaberkoUlrich MeschederJakub SorockiUlrik HankeJakub ZdartaUmamahesh BalijapalliJamal BalitiUmamaheswari RajajiJamal MabroukiUmar FarooqJamal NasirUmberto MichelucciJames Grant-JacobUrmita SikderJán GamecUsama HumayounJan HaidlUsha Devi YalavarthiJan HošekUsha Narayana PillaiJan KlimaszewskiUwe HartmannJán LabunUwe PliquettJan MikkelsenV. Indra GandhiJan PetrVadim SamarkinJanakarajan RamkumarVadim ShlyonskyJandas Ponnath JanardhananVaibhav HendreJanez TronteljVaibhav S. KathavateJang-Hsing HsiehVaidas PacebutasJanina AdamusValentina Di MeoJanira RoanaValentyn VolkovJanis AlnisValeria DemontisJannik LocklValeria ReginattoJanusz KluczynskiValeriano CorbelliniJanusz MikołajczykValerio Francesco AnneseJanusz NarkiewiczValerio PerenoJaroslav HrickoValery V. KalinchukJaroslaw W. KlosVallidevi KrishnamurthyJason BeechVamsi K. YadavalliJasti SateeshVan Son TrinhJavad KoohsorkhiVan Thanh NguyenJavad MohammadpourVan Thanh Tien NguyenJavanmardi SaeedVasileios PavlidisJaved SayyadVasily A. MilyutinJavier GarcíaVasily KosyanchukJavier Moreno-ValenzuelaVasyl MartsenyukJawad MirzaVeasna SoumJay AiraoVedat TopsakalJay GuoVeerakumar ChinnasamyJayashri KulkarniVenkata Siva BathulaJayer FernandesVenkatesan KalaichelviJayshri KulkarniVenkatesh ChenrayanJayu KalambeVenkatraju JellaJean ColombaniVeronica IacovacciJean-Baptiste LugagneVeronika KovacovaJean-Emmanuel BroquinVeta GhenescuJean-Pierre DelvilleVibhor KumarJean-Pierre HuignardViboon SaetangJeesu KimVicente AboitesJe-Kyun ParkVictor A. EremeyevJelena LazovicVictor Genchev IvanovJelena LillepärgVictor H. Perez-GonzalezJelena LovićVictor NazarychevJennifer BoothbyVíctor Ruiz-Valdepeñas MontielJennifer GerasimovVictor SongmeneJens-Peter ZöllnerVictor SverbilovJen-Yuan (James) ChangVictoria CorregidorJeong Hoon LeeVignesh ShanbhagJeongHun ParkVijay Bhooshan KumarJeongmoo HuhVijaya Gopalan SreeJerome ColinVijayakumar AnandJérôme Polesel-MarisVijayendra ShastriJeronimo Segovia-FernandezVi-Khanh TruongJerzy PotenckiViktor A. SoiferJesse RumboViktor LapshinJesus E. Velazquez-PerezVinay SinghJi FanVinaya kumar Kadayra BasavarajappaJia LiVincent MauriceJia LiuVincenzo RandazzoJiachou WangVinu MohanJia-De LinVipin AmoliJiahuan JiangVirendra Kumar YadavJiajun WuVirginija JankauskaiteJian ChengVisanu WanchaiJian Jian ChengViswanath Padmanabhan RameshJian LuVitali KononenkoJian PengVivek BajpaiJian ZhangVivek KumarJian ZhouVivek SaraswatJiandong WuVivekanandan RamanJianfei ChenVivekananthan VenkateswaranJianfei YinVječislav BohanekJiang WuVladimir B. ShirokovJiangfan YuVladimir BelyĭJiangguo ZhangVladimír CambelJianguo HuVladimír ChmelkoJianhui LiuVladimir Ivanovich VelkinJianjun LuoVladimir O. BessonovJiankang HuangVladimir PodolskiyJianlei WangVladimir S. LyubopytovJianliang XiaoVladimir SinitsinJianlong JiVladimir UlanskyJianming WeiVladimir V. AndreevJianping GuoVlasoula BekiariJianqiang YuVojko MatkoJianqiao HuVolker Paul SchulzJianyu FuVolker SchöppnerJianyun ShenVolodymyr SokolovJiaoqing PanVratislav FabianJiaqi HanVytautas OstaseviciusJiaqi LiuW. B. LeeJiaqi ZhuWahaj Abbas AwanJiaquan YuWahaj AwanJiawei WangWai Lun LoJiawen JianWaldemar WodziakJiawen XuWaleed Mohamed Abd-ElhameedJidesh P.Walid HassenJie DengWalter FuscaldoJie GaoWalter LangJie HuWan Hazman DanialJie YinWanbin RenJie ZhangWan-Chen HuangJie ZhouWanfeng ShangJifang WanWang GangfengJigang HuangWanting NiuJihoon KimWanyuan ShiJihoon SeoWaqas BangyalJihua ZhangWasna′a M. AbdulridhaJijian XuWei ChenJikai WangWei LiJik-Chang LeongWei LiangJilai WangWei LinJin LiWei WangJin MengWei WuJin XuWei YangJina KoWei YuanJinbo FeiWei ZhangJincan ZhangWei-Chiang HongJin-Chen HsuWeida HuJin-Cherng HsuWeiguo HuJing GuoWeihai HuangJing LiWeihe XuJing TianWeijiang XuJing WenWeijie TanJing XuWeijin GuoJingang LiWei-Kai ChengJingfeng HuangWeimin ChenJingjiang QiuWeipeng SunJinglian DuWeiqun LiuJingshu GuoWeishan ChenJingyu WangWeisong LingJingzhou ZhaoWeiwei ShiJinhua ZhouWeiyang QinJinhyoung ParkWeiying ChengJinny YeaahWeizhai BaoJinoop Arackal NarayananWen QinJinquan LiuWenbin ZhongJinsheng LuWenchao TianJin-Shuh LiWen-cheng KuoJin-Shyan LeeWendong WangJinsong WuWenfeng HaoJinsu GimWenfu ZhangJinwei CaoWen-Fung LiuJinyang XuWenkun XieJin-Young KimWenlong ChangJiří RezekWenlong LiJitendra PalWenqian ChenJitendra SinghWenqiang GuJiun Cai OngWen-Sheng ZhaoJiun-Ren HwangWenxiao ChuJiuren ZhouWenzhi CuiJiwoo HongWidiyastuti WidiyastutiJj ZhengWiesław P. JakubikJo VliegenWilfrido Calleja-ArriagaJoan BasWilfried WunderlichJoão M. MirandaWilliam Andrew SimonJoão MouroWilliam C. WilsonJoao Nuno MatosWilliam ChiappimJoão Paulo CarmoWilliam Edward LeeJoao Ricardo ReisWilliam ScheidelerJoaquim Miguel MaiaWin-jet LuoJochen FickWojciech WojtasiakJoe C. CampbellWolfgang HilberJoel DestinoWook KimJohann OsmaWoon Kiow LeeJohn AyeelyanWout De BackerJohn D. KechagiasWritam BanerjeeJohn H. MooreWujie ZhangJohn Jairo Villarejo MayorWujun ChenJohn M. AckenWu-Sung YaoJohn McGrathWuyi MingJohn N. RandallXavier ChiementinJohn P. XanthakisXavier FernandoJohn XuXi DengJojo JosephXiadong WangJolanta BiegańskaXian WuJon Gutiérrez EtxebarriaXiang LiJonathan F. HolzmanXiang LinJonathan PerreaultXiang QianJonathan SeyboldXiangkun YinJonathon TanksXianglong LiuJong Boon OoiXiangxi MengJong Youl ChoiXiangyu GaoJong-Chan KimXiangzhong ChenJonghyun OhXiangzhou LaoJong-Kwon LeeXianhao LeJong-Nam KimXianzheng ZongJongwan LeeXiao LiuJoo Hyun MoonXiao XiaoJoon Hyong ChoXiao ZhangJoonchul ShinXiaobin XiongJoong Ho ShinXiaodong ShiJoon-Hyuk ParkXiaofeng Allen SuJörg ImbrockXiaohui LiJorge Hernando-GarciaXiaojun WeiJorge Luis Camas AnzuetoXiaoliang ChenJorge MartinsXiaoliang LiangJorge Moreno RubioXiaomeng LiuJorge TrasobaresXiaomin LiuJose A. MoriñigoXiaoning ZhangJosé A. Siqueira DiasXiaopeng HaoJose AndradeXiaoqiang LiJosé BalsaXiaoShuai LiuJose C. S. CostaXiaowei YuJosé Higino CorreiaXiaowei ZhangJose I. PradoXiaoyang KangJosé Israel Martínez LópezXiaoye HuoJosé Jaime Taha-TijerinaXiaoyi LiJosé Jair Alves Mendes JuniorXiaoyu ChenJosé Luis Sánchez-RojasXiaoyu ZhangJose M. LlorensXiaozhong QuJosé Manuel Marques Martins De AlmeidaXichun LuoJosé Manuel QueroXin CuiJose Maria Flores AriasXin LuoJosé Martínez-LilloXin ShenJose Ortiz GonzalezXin YuanJosé R. LlataXin ZhaoJosé SaenzXin ZhouJosé ValdezXinbo ZouJose Vicente Abellan-NebotXinchen WuJose-maria CarazoXing GaoJoseph Chang Lun ChanXingfang LiuJoseph GovanXinghao HuJoseph LovecchioXinglai ZhangJosh PinskierXinglin YuJoshua BurrowXinglong DongJothiramalingam KulothunganXingpeng YanJozef BockoXinlei LiJozef DobranskyXinlin YanJózef DrewniakXinqi TianJozef KrajnakXinqin LiaoJozef SvetlíkXinxiang ZhangJózsef KutiXinyang SuJuan C. CruzXinyu XueJuan Carlos F. Rodriguez-ReyesXinyu ZhangJuan Ignacio Ahuir-TorresXinyue LiuJuan Monzó-CabreraXiongwei WuJuan MoralesXiuguo ChenJuan Pablo PascualXiyue ZhangJuan WangXuan LuoJuan ZhuXudong JiJuana Isabel MéndezXudong LinJuliana JohariXue HanJuliana SchellXuedan WuJulien MagnienXuefeng ZhuJulio PinheiroXuejing WangJuliusz LeszczynskiXuelin YangJun Yi Xuelu LiJun LiXue-Lun WangJun ShimizuXuezhi MaJun YangXuming ZhangJun ZhaXutao MeiJun ZhuXuye ZhuangJun-Eun ParkXuzhou LiJung-hong MinYa ZhangJunghoon YeomYabin JinJunghyeon OhYadie YangJungwon KangYael NemirovskyJunho ParkYagmur Demircan YalcinJun-Hwan ShinYakov RoizinJunjie LiYamin LiJunjie ZhangYan CaiJunkao LiuYan Jin Lionel LeeJunlei QiYan WangJunling HuYanal FaouriJunmei WuYanala Dharmendar ReddyJunmin LeeYanan YaoJunren WangYan-Cherng LinJunsheng LiangYancong QiaoJuntao LiuYanfeng WangJuntian QuYang Jun KangJunyong ParkYang LiJuraj JagelčákYang LiaoJustin B. KingYang LinJuvenal Rodríguez-ReséndizYang LiuJyoti JaiswalYang RanK. M. Faridul HasanYang ZhangKadir BilisikYanglong LuKah How KohYangmin LiKai ChengYangong ZhengKai KeYanhu ZhangKai LiYanhua ShaoKai OßwaldYanhua ZhaoKai Seng KohYaniv GelbsteinKai YuYanjun HanKai ZhaoYannick BleuKai-Da XuYanping XuKai-Ming HuYanquan GengKaiqiang QinYansheng LiangKairong QinYantao ShiKaixuan ZhangYao DuanKalyan Kumar JenaYao LiangKam NedevYao LuKamaljit RangraYao ZhaoKamel SultanYaobin TianKamil GocYaobing ZhaoKamil KarimullinYao-Feng ChangKan LiYao-Hsien LiuKan ShojiYaohua LiangKan WuYaoyu CaoKanak KalitaYasmin Mustapha KamilKang ZhouYasunori TakedaKangchun LeeYatao PengKang-Di LuYavuz ErtaşKannan Udaya MohananYe HeKaoru UesugiYe ZhangKari KopraYee Rui KohKarim MynbaevYee-wen YenKarina Suarez-AlcantaraYei Hwan JungKarla HillerYen-Wen LuKarolina Urszula LaszczykYeong-Her WangKarsten KöhlerYeonhoo KimKarthikeya Gulur SadanandaYeon-Mo YangKarunamoorthy SaravanakumarYeping HuKasumawati LiasYeu-Long JiangKatarina ItrićYi ChenKatarzyna GasYi GuKatarzyna KoziełYi HuangKatarzyna PetaYi LuKatarzyna TandeckaYi SunKaterina MouralovaYi WangKatharina VoelkelYi XuKátia FernandesYi ZhangKatrin SchmittYi ZhaoKatsuhiko ArigaYichao ZhaoKatsushi FurutaniYide ZhangKatsuyuki MachidaYiding LinKaupo KukliYifan LiKaveh MoulaeeYi-Fei WangKazi Fazle RabbiYih JengKazimierz FabisiakYih-Shing LeeKazunari KuritaYihui PanKazunari OzasaYi-Je JuangKazunari YoshidaYijun CaiKazuo OnoYi-Kuang YenKe PanYi-Lin YuKe TianYilun SunKeerti M. NaikYimin YangKefayat UllahYin FangKehao WangYing HongKeiji MurayamaYingchao YangKen SuzukiYing-Chih LiaoKenan LiYingWei FanKenji YoshinoYingxi XieKennedy O. OkokpujieYingxiang LiuKensuke OgawaYiqiang FanKentaro TotsuYiqiang ZhaoKerstin LängeYiwei HanKeun-Yeong JeongYiwei QiangKevin ChauYixiang DengKevin FiteYixing HuangKhadija MaqboolYiyu ZhangKhaled A. SallamYiyuan YangKhaled MnaymnehYogeenth KumaresanKhaled S. MekheimerYogendra Pratap SinghKhaled ZennirYogesh Kumar SrivastavaKhalil TamersitYogesh Kumar VermaKhalil Ur RehmanYohann ThimontKhurram Karim QureshiYoKo YamanishiKieu The Loan TrinhYong Jin JeongKiichi SatoYong LiKillol Vishnuprasad PandyaYong RenKim YeonginYong XiaoKiran Kumar TadiYong XuKirill Andreevich KuznetsovYong ZhangKirill GoncharYong ZhongKirill I. RybakovYongbo DengKirill LozovoyYonggang ZhuKirill V. HoroshenkovYonghai ZhangKishor Kumar GajraniYonghe MaKishore Kumar S.Yong-Hyuk MoonKishu RanjanYongjian LiKiverin AlexeyYongkun SuiKiwon ParkYongliang TangKiyotaka FujisakiYongqiang JiKlaudia KocurekYongqing HeKohei ToyodaYongsheng ZhouKoji MatsuuraYoon-Chul KimKoji NagataYoonseok ParkKonda Srinivasa RaoYooseob SongKonlin ShenYoshi MarienKonstantinos TatasYoshishige TsuchiyaKosmas L. TsakmakidisYoucef BelkhierKostiantyn TorokhtiiYounes AminiKosuke InoYoung Eun SongKosuke MinamiYoung Min SongKotaro MoriYoung Pyo JeonKotha GangadharYoung-Geun ParkKottilingam KottursamyYoung-hak ChoKourosh SheibaniYoung-Ho KoKris A. CampbellYounghyun KimKrishna KantYousef El HasadiKrishnendu BhattacharyyaYousif MohammedKristin HechtYu HuangKrzysztof CzubaYu Kyoung RyuKrzysztof DutkowskiYu Ting TsaiKrzysztof LalikYu ZhangKrzysztof PieńkowskiYu. M. BelousovKuanyu ChenYu. V. IoniKuldeep K. SaxenaYu.V. KnyazevKumi-Barimah EricYuan DongKumud Malika TripathiYuan GaoKundalwal ShaileshYuan WangKun-Lin LeeYuan XuKuntal MaityYuan YuanKuo-Sheng KaoYuan-Fong Chou ChauKushal SharmaYuan-Jen ChangKwang Hong LeeYuan-Pang HsiehKwangsoo KimYuanpeng WuKyohei HisanoYuansheng ZhouKyo-in KooYuanxi SunKyojiro MorikawaYuanyong LuoKyun Ho LeeYuanyuan DongKyun Kyu KimYucheng ChenKyung-A HyunYuchih ChenKyungwho ChoiYu-Chun KungL′uboslav StrakaYuehua LiLachit DuttaYueyang BenLadislau VekasYuezong WangLadislav VrsalovićYufei LiuLaibao ZhangYufeng QinLakshmi Narayanan GopalakrishnanYu-feng SuLal Mohan BhowmikYufeng WangLang ZhouYufeng ZhangLanju MeiYugang YuLara RebaioliYugang ZhaoLarisa StepanovaYuhao QiangLars SchönemannYuimaru KuboLászló Csurgai-HorváthYu-Jen WangLászló SzabóYu-Jui FanLatif AhmadYukang FengLaura AnfossiYuki OkamotoLaura BrattainYukui CaiLaurent LinguetYulia DavydovaLaurent RaymondYulin SongLaurent ThieryYulin WangLawrence C. Edomwonyi-OtuYuling NiuLazhar KherijiYulong SongLe WanYu-Lung LoLeandro GarcíaYung Hsiang ChenLeanne Lai-Hang ChanYung TingLechosław TuzYung-Shin SunLei FanYunjia LiLei PanYunpeng ShanLei ShaoYunqi CaoLei WangYunru YuLei ZhangYuqi LiLei ZhaoYuqian ZhangLeif KariYuri FilatovLeilei ShiYuri FreemanLeiming GaoYuri V. PetrovLena GolubewaYu-Sheng LinLeo AnzagiraYushun ZengLeon AbelmannYusuf Valentino KanetiLeonard AtanaseYuta TsujiLeonardo De Sousa SilvaYutao HuoLeonardo RundoYutaro AkimotoLeonardo Sastoque PinillaYuwei HuangLeonel Paredes-MadridYu-Wei SuLev A. MatveevYuxin LengLeyre CatalanYuxin XingLi JinYuxin ZhaoLi QiangYuya MorimotoLi ZhengYves BernardLian LiYves RoussignéLiang GaoZafer DursunkayaLiang JiangZahabul IslamLiang XieZaida OrtegaLiang ZhangZaka UllahLiangxing HuZakaria TagnamasLianqun ZhouZakriya MohammedLiao WuZaky A. ZakyLiaqat AliZan NieLibo ZhaoZbigniew BrytanLídia GonçalvesZbigniew HajdukLidia PutlyaevaZbigniew JaroszewiczLie WuZbigniew ZembatyLifeng QinZbynek RaidaLihong LiZdenek SoferLihua WangZebing MaoLihui HuangZecong FangLim ByeonghwaZedong LiLin BaoZeeshan AsgharLin ChengZeeshan KhanLin DongZehao HeLin GuiZehua LiuLin LiZeinab Malekshahi BeiranvandLin QiuZeljka PetrovicLin ZhangZeming SunLinchuan ZhaoZengxia MeiLinfei YinZesheng ChenLinghang WangZexi LiangLingju MengZhang LiuLingqian ZhangZhanghua HanLingxin ChenZhanqing YuLinhan LinZhanxiao KangLinh-An PhanZhao FengLino MisogutiZhao HaoLinyue TongZhaoguo XueLionel GamarraZhaohui WangLioua KolsiZhaoning YuLip Ket ChinZhaoshu YangLiqiang LuoZhaowei ChenLiqing RenZhaowei ZhangLiuan LiZhaoyan ShenLiudmila G. ShamanaevaZhe ShuLiuyang ZhangZheming ZhouLiwang LiuZhen QiuLi-Wei TsengZhen ZhangLiyun FanZheng LiLizhan ZengZheng YangLjubica KaziZheng ZhangLongchao ZhuoZheng Zhang Longju LiuZhenghong DaiLong-Sheng ChenZhengli HanLongshi RaoZhenglin YangLongxiang GuoZhengran HeLorand SzaboZhengsheng HanLorenzo BonettiZhenHua TangLou WenzhongZhenlin ZhangLouis Wy LiuZhenqiang YaoLu YinZhenqing LiuLuca BrunoZhenwei ZhangLuca La NotteZhenyin HaiLuca MenilliZhenyu ZhangLucas EncarnaçãoZheyuan ChenLucia BaldinoZhi QiaoLucia KnapcikovaZhi YanLucian TrifinaZhichao DongLuciano BoglioneZhifang ZhouLucjan SetlakZhifeng DingLudi MiaoZhifeng ZhangLuigi CampanellaZhifu LiuLuigi CordaroZhifu ZhangLuigi FortunaZhigang JiLuis Bernardo VarelaZhigang YinLuis CáceresZhiheng HuangLuis Castano LondonoZhijian SunLuis DuarteZhikang LiLuis Guerra RosaZhilai LuLuis LarreaZhilong SongLuis M. N. TavoraZhimin FanLuis Miguel Gomes AbegãoZhipeng ZhaoLuis N. López De LacalleZhiqiang LiangLuis RedondoZhiran YiLuisa FattorussoZhitian ShiLuisa PilanZhixiang HuangLuiz Antonio Alcântara PereiraZhixin CuiLuiz SantosZhiyu WangLukas BrauschZhiyuan ZhuŁukasz MajZhizhong TongLuke P. LeeZhongjing RenLulu WangZhongkai ChengLung-Chien ChenZhongke WangLuong-Viet PhungZhongming FanLuqman Hakim Ahmad ShahZhongrui WangLurui ZhaoZhongyuan FangM. L. MeenaZhou JianyuM. Raveendra KiranZhou ZhangMaawiya Ould SidiZhoufeng YingMadalina Simona BaltatuZhu DiaoMaddaka ReddeppaZhuoliang NiMadhan Kumar ArulanandamZhuo-Zhi ZhangMagda Latorre-EstevezZia SaadatniaMagdalena MatuszakZia Ullah KhanMagdalena Niemczewska-WójcikZiad SaghirMagdalena OsialZiang FengMahaveer KurkuriZifang ZhaoMahidur Rahman SarkerZihui WangMahmood Rafaei-BooketZining YangMahmoud ElsisiZinovi DashevskyMahmoud ZadehbagheriZiqi YuMahnaz NajafiZixiang Leonardo LiuMai The VuZiyang ZhangMaiara De Jesus BassiZlatin ZlatevMairtin O′DromaZofia RzepeckaMajid KhazaeeZohra BenzartiMajid MalboubiZong QinMajid MehrasaZongan LiMakoto MiyasakaZoran JakšićMałgorzata KowalczykZoran StamenkovicMallikarjuna MallappaZorana MilanovicMamadou Kaba TraoréZoubir MahdjoubManabu AtakaZouhaier MehrezManas Ranjan GartiaZuguang BianManasa KandulaZuomin Zhao

